# The Effect of Variability in the Powder/Liquid Ratio on the Strength of Zinc Phosphate Cement

**DOI:** 10.1155/2011/679315

**Published:** 2011-12-08

**Authors:** Jill E. McKenna, Noel J. Ray, Gerald McKenna, Francis M. Burke

**Affiliations:** Restorative Dentistry, Cork University Dental School and Hospital, University College Cork, Ireland

## Abstract

*Aim*. To investigate (a) variability in powder/liquid proportioning and (b) effect of variability on diametral tensile strength (DTS), in a zinc phosphate cement. Statistical analyses (*α* = 0.05) were by Student's *t*-test in the case of powder/liquid ratio and one-way ANOVA and Tukey HSD for pair-wise comparisons of mean DTS. The Null hypotheses were that (a) the powder-liquid mixing ratios would not differ from the manufacturer's recommended ratio (b) DTS of the set cement samples using the extreme powder/liquid ratios would not differ from those made using the recommended ratio. *Methodology*. 34 dental students dispensed the components according to the manufacturer's instructions. The maximum and minimum powder/liquid ratios, together with the manufacturer's recommended ratio, were used to prepare samples for DTS testing. *Results*. Powder/liquid ratios ranged from 2.386 to 1.018. The mean ratio (1.644) was not significantly different from the recommended value of 1.718 (*P* = 0.189). DTS values for the maximum and minimum ratios were both significantly different from each other (*P* < 0.001) and from the mean value obtained from the recommended ratio (*P* < 0.001). *Conclusions*. Variability exists in powder/liquid ratio for hand dispensed zinc phosphate cement. This variability can affect the DTS of the set material.

## 1. Introduction

Indirect restorations play an important role in clinical dentistry. Whilst clinicians often spend considerable time choosing materials or type of restorations, the cementation process is often considered less important and manufacturer's instructions modified or ignored [1]. Luting materials are necessary to retain such restorations on teeth and to prevent leakage at their margins [2]. One such material is zinc phosphate cement which is used routinely by almost one-third of UK practitioners [3].

Clinically, the cement is presented as powder and liquid components which must be mixed together prior to application to the restoration or the tooth. Often the proportions of the two elements used are measured by eye or with operator-dependent measuring systems. They are then mixed together in varying environments (temperature and humidity) under differing mixing conditions—time and manipulation technique [4]. While low-grade mixing errors (<10–20%) may not have serious implications regarding the integrity of traditional luting agents, there is concern regarding high-grade errors [1]. Such errors are likely to occur with less experienced operators, such as undergraduate dental students and inexperienced dental nurses.

As a measure of mechanical integrity of the set cement, compressive strength has been found to increase with the amount of powder mixed with a constant amount of liquid [4–8]. In addition, diametral tensile strength (DTS) is a well-established and reproducible measure of mechanical integrity in very brittle materials such as zinc phosphate cement and has been extensively used in the testing of appropriate dentally related materials [9–11].

The aim of this study was to (a) investigate whether high-grade powder-liquid proportioning errors might occur in a cohort of inexperienced dental undergraduate students mixing zinc phosphate cement and (b) determine the effect of any such variability on the diametral tensile strength of the set material. The Null hypotheses were that (a) the powder-liquid mixing ratios observed would not differ from the manufacturer's recommended ratio and (b) DTS of the set cement samples using the extreme powder/liquid ratios observed would not differ from those made using the manufacturer's recommended ratio.

## 2. Methodology

The entire third year undergraduate dentistry class from a dental school in Ireland was invited to participate in the study. Thirty four students contributed to the experiment, none of whom had previous experience of working with zinc phosphate cement but had experience of mixing other dental materials including glass ionomer cement in the clinical environment. Each student received verbal and written directions in measuring powder and liquid components according to the manufacturer's instructions (Fleck's Cement, Mizzy, Cherry Hill, NJ, USA). The instructions stated that 0.8 g of powder should equate with “2 fillings to the shoulder of the powder cap dome” and 0.3 mL liquid should equate with “12 drops of liquid” (luting ratio). The students measured the powder and liquid using the powder cap and liquid dropper onto a preweighed plastic container and the mass of each was recorded (Mettler AE 100 balance, Mettler Instruments AG, Greifensee, Zurich). A calibrated pipette was used to determine the density of the cement liquid yielding the mass equivalent of the manufacturer's recommended volume of 0.3 mLs and a powder/liquid ratio (m/m) of 1.718.

The observed maximum (2.386) and minimum (1.018) powder/liquid ratios, together with the manufacturer's recommended ratio (m/m), were used subsequently by a single experienced dental clinician to prepare cylindrical samples (*n* = 3 × 34), of nominal dimensions 6 mm × 3 mm, using a stainless steel split mould. The samples were hand mixed on a chilled glass slab and the mix transferred and packed into the split mould. The samples were left to initially mature (60 mins) before being removed from the mould. Excess material was removed using a sharp scalpel. The specimens were further matured in deionised water (37 ± 2)°C for 48 hours. Before testing, each specimen was dried gently on all sides using soft tissue paper and the diameter and height measured using a micrometer gauge. Compression across a diameter was carried out using a universal testing machine, H10KS (Tinius Olsen), at a constant crosshead speed of 0.75 mm/min. Failure load data were converted to DTS using the expression *σ* = 2*P*/*πdl* (*σ* = diametral tensile strength, *P* = load at failure, *d*  =  diameter of cylinder, and *l* = height of cylinder). All testing was at 23 ± 2°C.

The Null hypotheses were that (a) the powder-liquid mixing ratios observed would not differ from the manufacturer's recommended ratio and (b) DTS of the set cement samples would not differ from those made using the manufacturer's recommended ratio. Statistical analyses (*α* = 0.05) were by Student's *t*-test in the case of powder/liquid ratio and one-way ANOVA and Tukey HSD for for pair-wise comparisons of mean DTS (R version 2.8.0—R Foundation for Statistical Computing). Appropriate tests were carried out for normality and constant variability.

## 3. Results

The extreme powder/liquid mixing ratios (m/m) observed from the student cohort, together with the manufacturer's recommending ratio, and corresponding data regarding DTS are presented in Table 1. The mean powder/liquid ratio obtained for the student cohort was not significantly different from the manufacturer's recommended value (*P* = 0.189). The mean DTS values recorded for the maximum and minimum ratios for the student cohort were significantly different from that obtained from the manufacturer's recommended powder/liquid ratio (*P* < 0.001) and from each other (*P* < 0.001).

## 4. Discussion

It is clear that high-grade powder-liquid proportioning errors were observed in this cohort of dental undergraduate students in the sense that the extreme powder-liquid ratios resulted in significant impacts on DTS. These proportioning errors have occurred notwithstanding explicit verbal and written instructions given immediately before the mixing process. While it is attractive to attribute this effect to “inexperience,” as might be relevant to undergraduate dental students and inexperienced dental nursing staff, more substantial and quantifiable concerns must address the mixing methodology. Ideally, the mixing process should be “fail safe” in that it should only be possible to mix material correctly, or not at all. In this regard, a number of opportunities for error present.

The volume of powder delivered using volume dispensation is very operator dependent and variations can be common due to the differences in cement powder packing densities when filling the scoop [12, 13]. For dispensing the liquid the opportunity for inaccuracy is potentially even greater. The amount of liquid expressed is dependent on the manner in which the bottle is held and the force exerted on it. Excessive force can cause a stream of liquid to be expressed rather than a series of drops and air bubbles can further complicate measurement [13]. Operators often tend to mix cements and other restorative materials by “experience”, adding extra powder or liquid to gain the consistency or properties they desire [14]. A report quoted relatively good intraoperator consistency in mixing zinc phosphate cement (single commercial product) among a cohort of 40 qualified dental nurses in the UK but little intra-operator consistency [15]. Clearly, such a report is not conducive to the expectation of acceptable performance among inexperienced operators and begs the overall question of whether an alternative approach should be used in the manner of a standard operating protocol per product. A plausible solution to addressing variability in powder/liquid ratio (and presenting the possibility of consistent mixing) is the encapsulation of cement components (single-use capsules) which certain manufacturers have done with zinc phosphate and other luting cements such as glass ionomer [16]. Encapsulation would also remove the problem of solvent evaporation from the cement liquid with possible phosphate deposition yielding the liquid unusable.

It is, however, unclear what parameters the manufacturer's recommended powder/liquid address. Presumably, the resultant mixture is required to be sufficiently thin to wet the adherend but with the ability to yield acceptable strength on setting. A report on the effect of powder/liquid ratio on the clinical performance of resin-modified glass ionomer cement [17] indicates that higher powder/liquid ratios compromise surface wetting with an increased incidence of subsequent retention failures when used as a tooth restorative.

The reported mean value for DTS, mixed according to the manufacturer's ratio, was generally consistent with previously reported values for other brands of zinc phosphate cement relating to similar specimen dimensions and storage [18, 19]. This would suggest that the observed low value obtained from the minimum powder/liquid ratio may be generalised as inappropriate proportioning. The value was less than 38% of the strength associated with the manufacturer's mixing ratio and must raise concerns regarding mechanical adequacy of the set material. Apart from concerns regarding compromised mechanical properties, there are likely clinical consequences to the use of an inappropriate powder to liquid ratio. For example, incorporation of too much powder into the luting cement may result in a thick mix of cement which could make complete seating an indirect restoration difficult [20]. This could result in open margins and/or a high restoration which could cause patient discomfort in the short term and the failure of the restoration in the long term.

While the use of encapsulated products might appear attractive to obviate arbitrary mixing ratios with attendant problems, a reported comparison of an encapsulated zinc phosphate cement with a hand-mixed analogue highlighted certain deficiencies in the encapsulated material [21, 22] No significant advantages in terms of reliability or strength were observed in the encapsulated product. In addition, the latter was observed to be more porous than the hand-mixed material, most likely due to the entrapment of air during the relatively vigorous mechanical trituration.

## 5. Conclusions

Null hypotheses (a) and (b) are rejected. High-grade proportioning errors relating to Fleck's Cement, for a luting consistency, were observed among a cohort of dental undergraduate students. These errors resulted in significant differences between the DTS values of the set cement and those which relate to the manufacturer's recommended ratio. Apart from compromised mechanical performance resulting from inappropriate proportioning, incorporation of too much powder into the luting cement may result in a thick mix of cement which could make complete seating an indirect restoration difficult. This could result in open margins and/or a high restoration. Routine encapsulation of zinc phosphate cement components, in the form of single-use disposable capsules, is suggested as a method of addressing variability in proportioning powder-liquid cements especially amongst inexperienced operators.

## Figures and Tables

**Table 1:** Extreme powder/liquid mixing ratios & corresponding mean diametral tensile strength.

	Maximum	Minimum	Mean	Manufacturer's
Powder/liquid ratio, m/m (SD)	2.386	1.018	1.644 (341)	1.718
Diametral tensile strength, MPa (SD)	7.19 (1.50)	2.65 (1.01)	—	6.01 (1.30)
